# Reconstructing charge-carrier dynamics in porous silicon membranes from time-resolved interferometric measurements

**DOI:** 10.1038/s41598-018-35210-z

**Published:** 2018-11-21

**Authors:** Wei He, Rihan Wu, Igor V. Yurkevich, Leigh T. Canham, Andrey Kaplan

**Affiliations:** 1 College of Physics and Materials Science, Henan Normal University, Xinxiang, 453007 People’s Republic of China; 2 School of Physics and Astronomy, University of Birmingham, Birmingham, B15 2TT United Kingdom; 3 Nonlinearity and Complexity Research Group, Aston University, Birmingham, B4 7ET United Kingdom

## Abstract

We performed interferometric time-resolved simultaneous reflectance and transmittance measurements to investigate the carrier dynamics in pump-probe experiments on thin porous silicon membranes. The experimental data was analysed by using a method built on the Wentzel-Kramers-Brillouin approximation and the Drude model, allowing us to reconstruct the excited carriers’ non-uniform distribution in space and its evolution in time. The analysis revealed that the carrier dynamics in porous silicon, with ~50% porosity and native oxide chemistry, is governed by the Shockley-Read-Hall recombination process with a characteristic time constant of 375 picoseconds, whereas diffusion makes an insignificant contribution as it is suppressed by the high rate of scattering.

## Introduction

Since the 1990s, research interest in Porous Silicon (*p*-Si) has grown considerably, following reports that showed photoluminescence in the visible range^1,2^. Soon thereafter, research into this sponge-like material exploded as it seemingly offered new directions for the development of a wide range of optical and electro-optical applications such as optical interference filters^3^, solar panel enhancers^4^, all-optical modulator for far-IR^5^, multilayer periodic structure with photonic gap^6^, electroluminescence material^7^, Mach-Zehnder interferometer based sensors^8^, and optical biosensors^9^.

One of the distinctive features of *p*-Si is its rigid sponge-like skeleton made of silicon having nano-metric dimensions. The specific surface area of *p*-Si can reach 800 m^2^/cm^2^, since porosity significantly enlarges its surface-to-volume ratio^10^. This property provides a new potential for photoluminescence and light trapping^11–14^. To improve the efficiency of *p*-Si based electronic and electro-optical devices, greater understanding and control of the charge transport mechanism and recombination dynamics is needed. For example, improved transport is crucial for photovoltaic devices to allow the photo-excited carriers to reach electrodes before recombination happens. It is preferable for such devices to establish conditions which combine the provision of charge with a long life time and a high diffusion coefficient^15,16^. On the other hand, a fast relaxation into the conduction band minimum and recombination with holes is critical for the high performance of light modulation devices^17,18^. The investigation of these transport properties is a complex task as it involves a number of phenomena, such as scattering, recombination and diffusion, which are usually difficult to characterise in a single measurement. The most common experimental methods for investigating charge carrier transport is to measure current-voltage (I-V) characteristics or steady-state photoconductivity. However, the problem of fabrication of reproducible, low resistivity and stable contacts on a *p*-Si surface has probably influenced most studies to date^19^. Moreover, in many cases, *p*-Si samples are usually composed of a porous silicon layer on top of a crystalline silicon (c-Si) substrate, which might distort accurate estimation of the transport properties^20–22^. To avoid these problems in this work, we used free-standing thin membranes of *p*-Si and contactless ultrafast optical methods.

The main purpose of this work is to establish, as unambiguously as possible, the carrier dynamics and related constants of the moderately mesoporous material, with a stabilised native oxide protective shell. We used the time-resolved femtosecond pump-probe method to simultaneously measure reflectance and transmittance spectra in the temporal range up to 210 ps after the excitation by a femtosecond laser pulse. This experimental method uses the simultaneous recording of the time-resolved reflectance and transmittance interference fringes over the probe beam spectral range of 60 nm interacting with a thin optical *p*-Si membrane slab. The observation of the interferograms is not accidental, but had a deliberate purpose to select the thicknesses of membrane providing a high fringe contrast. These well-resolved fringes enhance the optical response of the probe interacting with the optically excited membrane and improve the sensitivity of the experiment; while, without the fringes on thick membranes, the change of the reflectance and transmittance induced by the pump could be too weak to successfully analyse.

Reflectance and transmittance were recorded simultaneously as a function of the wavelength in order to reduce the number of free parameters and increase the fidelity in the simulation of the experimental data. For the experimental data analysis, we used a recently developed method based on the Wentzel-Kramers-Brillouin (WKB) approximation^23,24^. Using this method, we retrieved a non-uniform spatial distribution of the excited charge carriers and their evolution as a function of time. We show that the carrier dynamics in our samples are governed exclusively by the recombination process, whereas the contribution of the diffusion is insignificant. From our measurements, we estimated the recombination time to be 375 ps.

## Results

### Time resolved pump probe measurements and analysis

**Figure 1:**
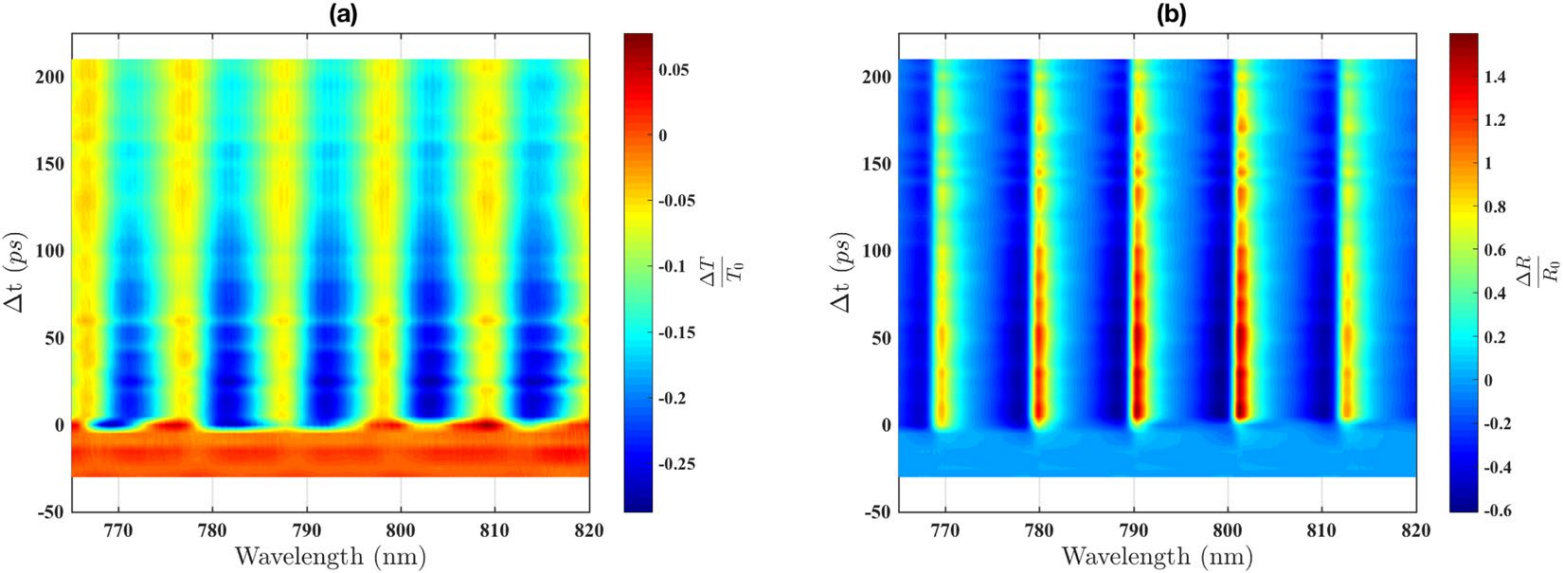
Simultaneously recorded fractional change of the transmittance (**a**), Δ*T*/*T*_0_, and reflectance (**b**), Δ*R*/*R*_0_, as a function of the delay time, Δ*t*, and probe wavelength.

To evaluate the excited carriers dynamics in the *p*-Si membrane, the time resolved pump-probe transmission and reflection were measured simultaneously over the wavelength range between 765 and 820 nm. The pump fluence was fixed at about 1.5 mJ/cm^2^. The time delay between the pump and probe was scanned from −20 to 210 ps, with 5 ps step size. Figure 1 shows the measurement results of Δ*T*/*T*_0_ and Δ*R*/*R*_0_ on the left and right panels, respectively. The signal at the negative delay times is set for the false-colour representation of the background values of Δ*T*/*T*_0_ and Δ*R*/*R*_0_. It can be seen that at the positive delay times both signals, Δ*T*/*T*_0_ and Δ*R*/*R*_0_, oscillate as a function of the wavelength. These oscillations represent the Fabry-Perot interference fringes of the probe beam propagating through the membrane while excited by the pump. The reason for the pump-induced fringes is the modification of the membrane dielectric function by the free carriers excited by the pump. This creates conditions at which the probe beam components, partially reflected and transmitted by the upper and lower boundaries of the membrane, interfere with one another and intensify or reduce the amount of reflected or transmitted light. The use of a thin membrane allows sensitivity of small (Δ*ε*/*ε* < 10^−3^) pump-induced changes of the dielectric function, through the measurements of the optical interference fringes in reflectance and transmittance spectra, and analysis using the thin-film optics equations^23,25,26^. The decrease of the fringe contrast as a function of time is related to the decay of the excitation when the dielectric function which is altered by the pump returns to the initial value. Thus, simulating the fringes with a suitable optical model allows for the retrieval of the complex dielectric function and its evolution as a function of time. Once the dielectric function evolution is obtained, it can be used to reconstruct the corresponding development of the carrier density using a high-frequency conductivity model, such as Drude theory^24,27^.

To model the optical response of the *p*-Si membrane, it was considered as a uniform homogeneously mixed material^5,27,28^, consisting of silicon matrix skeleton and pores filled with air. The optical response of the material can be represented by the effective dielectric function *ε*_*eff*_ of a 2D composite material described by the following Maxwell-Garnett formula^29,30^:

1 $${\varepsilon }_{eff}={\varepsilon }_{m}+2p{\varepsilon }_{m}\frac{{\varepsilon }_{p}-{\varepsilon }_{m}}{{\varepsilon }_{p}+{\varepsilon }_{m}-p({\varepsilon }_{p}-{\varepsilon }_{m})},$$

where *ε*_*m*_ and *ε*_*p*_ represent the dielectric functions of the membrane constituents, silicon and air pores, respectively, and *p* is the volume fraction of the pores. The pores were assumed to be a dispersionless material with *ε*_*p*_ = 1, dielectrically softer than the silicon skeleton constituent. Since the diameter of the pores and silicon constituents are significantly smaller than the probe wavelength, the assumption of the homogeneous medium approximation and the application of the Maxwell-Garnett formulas are valid^31,32^.

The free carriers excited optically by the pump modify the dielectric function of the silicon skeleton according to the Drude theory of high frequency conductivity^26,33,34^:

2 $${\varepsilon }_{m}={\varepsilon }_{{Si}}-\frac{{\omega }_{p}^{2}}{{\omega }^{2}+i\omega \gamma },$$

where

3 $${\omega }_{p}^{2}=\frac{{e}^{2}}{{\varepsilon }_{0}}(\frac{N}{{m}_{eff}})$$

represents the plasma frequency and *ω* is the probe frequency; *m*_*eff*_ = 0.17 is the reduced mass of the optically excited electron-hole plasma^27,35^; *e* is the electron charge and *ε*_0_ is the vacuum permittivity; *γ* denotes the charge-carrier scattering rate; *N* is the density of the free carriers excited by the pump; *ε*_*Si*_ is the complex dielectric function of the crystalline silicon used to fabricate the samples and whose value was determined previously^23^. The combinations of Eqs 2 and 3 substituted into Eq. 1 leads to the dependence of the effective dielectric function, *ε*_*eff*_, on the probe frequency, *ω*, scattering rate, *γ*, and the free carrier density, *N*. The decay of *N*(Δ*t*) induces changes to *ε*_*eff*_ (Δ*t*) and, consequently, to the contrast of fringes of Δ*T*/*T*_0_(Δ*t*) and Δ*R*/*R*_0_(Δ*t*). To fully incorporate the excited carrier density decay into the optical model, it must also be considered as a function of the sample depth *z*. Assuming that the pump is linearly absorbed, immediately after the excitation, when the pump and probe temporally overlap, this function can be presented as $N({\rm{\Delta }}t=0,z)={N}^{0}({\rm{\Delta }}t=\mathrm{0)}{e}^{-{\alpha }_{pump}z}$, where *α*_*pump*_ = 450 cm^−1^ is the effective absorption coefficient of the pump, which includes the absorption due to the properties of the material and internal multiple reflection from the sample boundaries; *N*^0^(Δ*t* = 0) is the excited carrier density on the sample surface at the zero delay. Therefore, the optical model calculating Δ*T*/*T*_0_(Δ*t*) and Δ*R*/*R*_0_(Δ*t*) is underpinned by the development of the carrier density in time and space, *N*(Δ*t*,*z*). The same argument applies to the effective dielectric function, *ε*_*eff*_ (Δ*t*,*z*) evolution in space and time. We note that, the complexity of the spatial development in time of the carrier density is somewhat relaxed in this work as the samples are quasi-one-dimensional, restricting the carrier movement along the interwoven wires of porous silicon.

To account for non-uniform *ε*_*eff*_ (*z*), in calculations of the transmittance and reflectance we used a method based on Wentzel-Kramers-Brillouin (WKB) approximation^36^, which was developed and previously used in our works on similar porous silicon membranes but having different porosity^23,24^:

4 $$T={|\sqrt{\frac{q\mathrm{(0)}}{q(d)}}\frac{\mathrm{(1}+r\mathrm{(0)(1}-r(d))}{{e}^{-i\psi }-r\mathrm{(0)}r(d){e}^{i\psi }}|}^{2}$$

5 $$R={|\frac{r\mathrm{(0)}{e}^{-i\psi }-r(d){e}^{i\psi }}{{e}^{-i\psi }-r\mathrm{(0)}r(d){e}^{i\psi }}|}^{2},$$

where *r*(0) and *r*(*d*) are the reflection coefficients of the front and rear sample boundaries, respectively; $q(z)=\sqrt{\frac{{\omega }^{2}}{{c}^{2}}\varepsilon {\rm{e}}{\rm{f}}{\rm{f}}(z)-{k}_{x}^{2}}$ is the wavevector of the probe along the *z* coordinate; ${k}_{x}=\frac{\omega }{c}sin(\theta )$ is the tangential component; *θ* is the incidence angle; $\psi ={\int }_{0}^{d}\,dzq(z)$ is the cumulative complex phase of the probe traversing the sample having thickness *d*.

To perform the calculation, it was estimated that ${N}^{0}({\rm{\Delta }}t=\mathrm{0)}=\mathrm{(1}-{R}_{0})\frac{F}{\hslash \omega }{\alpha }_{pump}=2\times {10}^{19}$ cm^−3^ and the corresponding scattering rate was taken from our previous work^5^ *γ*^0^(Δ*t* = 0) = 7 × 10^14^ s^−1^. These values were used to estimate *ε*_*eff*_ (*z*), which determines the Fresnel coefficients *r*(0) and *r*(*d*), the wavevectors *q*(0) and *q*(*d*), the phase *ψ* at the zero delay time. Then a generic algorithm was used iteratively to find the best function of *N*(Δ*t*,*z*) describing the experimentally measured change of the reflectance and transmittance, Δ*T*/*T*_0_(Δ*t*) and Δ*R*/*R*_0_(Δ*t*), for each delay time, Δ*t*. The scattering constant, *γ*, was readjusted to 6.6 × 10^14^ sec^−1^ to fit the data in the best way and it was kept constant as a function of time and space. We found that the alteration of *γ* has a relatively weak effect on the simulation, suggesting that the carrier relaxation rate is saturated under these experimental conditions.

**Figure 2:**
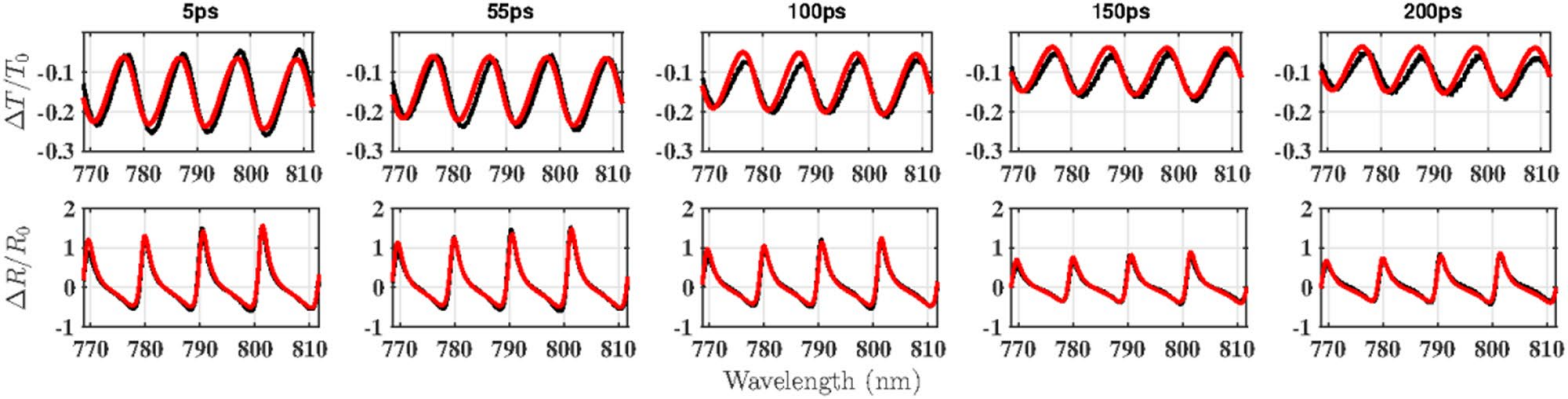
Representative transient spectra of the transmittance change, Δ*T*/*T*_0_ (top row), and reflectance change, Δ*R*/*R*_0_ (bottom), for the delay times of 5, 55, 100, 150 and 200 ps; black - experimental data and red - theoretical simulation.

To illustrate the fitting results, the data at several different time delay – 5, 55, 100, 150 and 200 ps–were picked out and shown in Fig. 2. It can be seen that the amplitudes of the fringes, shown as black dotted lines, gradually becomes weaker, and the fitting results, displayed as solid red lines, are a reasonable match for the data. Discrepancies of fit for Δ*T*/*T*_0_ at longer delay times were difficult to resolve without changing the model, but these are not significant and are tolerable without altering our interpretation of the results.

**Figure 3:**
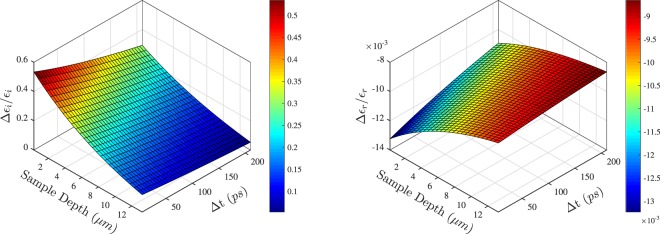
Fractional change of the imaginary, Δ*ε*_*i*_/*ε*_*i*_ (left panel), and real, Δ*ε*_*r*_/*ε*_*r*_ (right panel), parts of the effective dielectric constant as a function of the sample depth and delay time, Δ*t*.

The fractional changes of the real and imaginary parts of the dielectric function, Δ*ε*_*r*_/*ε*_*r*_ and Δ*ε*_*i*_/*ε*_*i*_, respectively, are shown in Fig. 3. Significantly, the change of the imaginary part is by two orders of magnitude greater than that of the real part. This is consistent with the evolution of the shape of the fringes observed in Figs 1, 2. However, the spectral positions of the maxima and minima of the fringes, governed by the real part of the dielectric function, do not change. In contract to this, is the width of the troughs and peaks, controlled mostly by the imaginary part, which noticeably decreases as the excitation decays as a function of both time and depth. Indeed, such behaviour is expected for a material where *γ *~ *ω*_*p*_ < *ω* and for which the Drude model predicts that the imaginary part can be approximated as ${\rm{\Delta }}{\varepsilon }_{i}\approx {\omega }_{p}^{2}\gamma /{\omega }^{3}$, while the real part is nearly constant^37^. In such conditions, the fractional change of the imaginary part, Δ*ε*_*i*_/*ε*_*i*_, depends linearly on the free carrier density, *N*, and its change almost exclusively governs the observed changes of the reflectance and transmittance, Δ*T*/*T*_0_ and Δ*R*/*R*_0_, respectively. In fact, their change can be exclusively attributed to the induced by pump free carrier absorption of the probe.

### Solving the carrier dynamics

**Figure 4:**
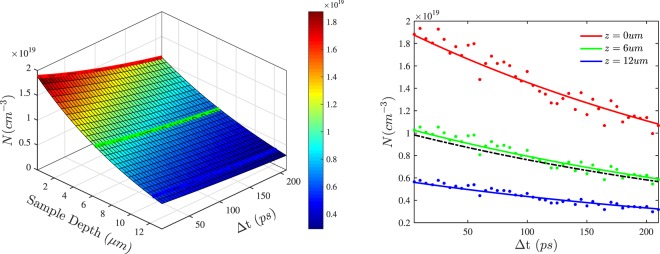
Left panel: reconstructed decay of the charge carrier density, *N*, inside silicon constituent of *p*-Si as a function of the sample depth and delay time, Δ*t*. Right panel: representative decay curves of the charge carrier density, *N*, at three different distances from the sample surface; red - on the surface: *z* = 0, green - *z* = 6 and blue - *z* = 12 *μ*m. Solid lines represent the model best-fitted to the experimental data, which are shown as dots. For convenience, the same lines are shown on the left panel as well. The black dashed line is the average carrier density, 〈*N*(*t*)〉.

The free carrier density reconstructed from the simulation, *N*(Δ*t*,*z*), is shown in Fig. 4. It can be seen that, to a large extent, it replicates the same trend shown in Fig. 3. *N*(Δ*t*,*z*) function can be used to evaluate the dynamics of the free carriers using the following rate equation:

6 $$\frac{dN(z,t)}{dt}=D\frac{{\partial }^{2}N(z,t)}{\partial {z}^{2}}-\frac{N(z,t)}{\tau }$$

In Eq. 6 the first term describes the one-dimensional diffusion along the depth coordinate, with *D* being the diffusion coefficient. In general, the diffusion is a three-dimensional process, but in our samples of porous silicon, consisting of wires nearly aligned along the depth coordinate, z, the process can be assumed as limited to one dimension only. The second term gives the recombination rate, 1/*τ*. In semiconductors, the recombination time, *τ*, can be dependant on *N*. The most common processes are Shockley-Read-Hall (SRH), bimolecular and Auger recombinations, where 1/*τ* is independent, linearly and quadratically dependant on *N*, respectively^38^. In samples with a non-uniform carrier distribution, the determination of the prevalent recombination process can be quite a complex task: as *τ*, might be spatially non-uniform because of its dependance on *N*. To resolve possible complications, we initially excluded the diffusion process from the rate equation. The average carrier density was estimated using $\langle N(t)\rangle =\mathrm{1/}d{\int }_{0}^{d}\,dzN(z,t)$, but in the integration, the diffusion term vanished, owing to zero-current boundary conditions at the edges of the sample. 〈*N*(*t*)〉 is shown in Fig. 4 along with the decay curves of the carrier density on the surface, *z* = 0, at the middle, *z* = 6 *μ*m, and near the rear boundary, *z* = 12 *μ*m, of the sample. All shown curves can be fitted by solving the ordinary differential equation $\frac{dN(z;t)}{dt}=-\frac{N(z;t)}{\tau }$ with *τ* = 3.75 ± 0.15 × 10^−10^ seconds. This suggests that SRH is the main form of recombination and that diffusion is a much slower process, having no observable impact on the carrier dynamics. Indeed, to observe the diffusion, the inequality $D\ge \mathrm{1/}{\alpha }_{pump}^{2}\tau$ must hold. However, the samples investigated had such a fast recombination that this was nearly impossible. Therefore, we conclude that the main recombination process is independent of carrier density and that the diffusion is effectively absent in the samples.

The origin of the SRH recombination is determined by the capture of carriers by the boron dopants and impurity states on the surface of the pores. The effective cross-section can be estimated according to *σ* = 1/*N*_*imp*_*vτ*, where *v* = (*k*_*B*_*T*/*m*)^1/2^ = 1.64 × 10^7^ cm/s is the carriers thermal velocity at room temperature. For the impurity density of *N*_*imp*_ = 3 × 10^18^ cm^−3^, the cross-section is *σ* = 5.42 × 10^−17^ cm^2^, a typical value for silicon at room temperature which has been known for decades^39^. This observation suggests that the SRH in *p*-Si does not significantly deviate from the bulk counterpart, as suggested previously^24^.

## Conclusions

In conclusion, we investigated the carrier dynamics using time resolved reflectance and transmittance interferograms of the probe beam. We applied the analysis based on the approximation of the *p*-Si sample as an effective medium described by 2D Maxwell-Garnett approximation. The contribution of the free carriers to the optical response was described by Drude theory, and their non-uniform distribution by Wentzel-Kramers-Brillouin approximation. Our study reveals that the carriers dynamics are outweighed by recombination, while the diffusion is undetectable. The simulation suggests that the excited carriers scattering time is *γ* = 6.6 × 10^14^ s^−1^, implying that the diffusion constant $D=\frac{{k}_{B}T}{{m}_{eff}}\frac{1}{\gamma } \sim 0.4$ cm^2^/s is much smaller than the recombination parameter $1/{\alpha }_{pump}^{2}\tau =1.32\times {10}^{4}$ cm^2^/s. The excited carrier spatial distribution and its decay, obtained by the experiment, indicate that the recombination time is independent of the carrier density, as would be expected for the Shockley-Read-Hall mechanism. We estimate the recombination time to be *τ* = 3.75 ± 0.15 × 10^−10^ seconds.

## Methods

### Time-resolved interferometric measurements

A Coherent ultrafast laser system was used for the femtosecond pump-probe setup. The system delivers 60-fs pulses at the repetition rate of 1 kHz and has an almost Gaussian-shaped spectrum centered around 795 nm. A beam splitter was used to split the laser into two parts: the pump and probe beams. The power ratio between the pump and probe was more than 100:1. A retroreflector delay stage was used to control the difference between the arrival times of the pump and probe pulses. A combination of a half-wave plate and Brewster angle reflection from a glass block was used to adjust the pump fluence. The polarization of the probe beam was adjusted to yield equal contributions of *s* and *p* components, while the pump beam was orthogonally polarized with respect to the probe beam to prevent interference between them. The incident angle of the probe beam was set to 45° and the angle difference between the pump and probe beams was ~20°. The probe and pump beam were focused to spot diameters of ~100 and ~300 *μ*m, respectively, by using different focusing lenses. The noncollinear spatial overlap between the pump and probe spots was checked by a CCD camera equipped with a magnifying lens. The temporal overlap between the pump and probe pulses was identified by second-harmonic generation from a BBO crystal positioned at the sample position. The intensities of the reflected and transmitted probe beam were wavelength analyzed by two spectrometers of the same type (Ocean Optics QE65 Pro). The detected data were presented in the form of a fractional change of the reflectance and transmittance^23^: Δ*R*/*R*_0_ = (*R*_*t*_ − *R*_0_)/*R*_0_ and Δ*T*/*T*_0_ = (*T*_*t*_ − *T*_0_)/*T*_0_, where *R*_*t*_ and *T*_*t*_ are the reflectance and transmittance of the excited-state sample at a time delay *t* after the pump excitation, respectively, and *R*_0_ and *T*_0_ are the reflectance and transmittance of the sample without excitation. More details of the experimental setup, data analysis and measurements of *R*_0_ and *T*_0_ can be found elsewhere^23,27,40^.

### Sample fabrication and characterisation

The investigated *p*-Si samples were fabricated by the electrochemical anodization of the surface of a 3′′-diameter (100) silicon wafer (Boron-doped, 5–15 mΩ cm, corresponding to the dopant’s density of ~3 × 10^18^ cm^−3^), using an electrolyte comprised of methanol and 40% HF in a 1:1 ratio. A current density of 30 mA/cm^2^ and an anodization time of 11 min were chosen to yield a layer with ~50% porosity (calculated by using a gravimetric calibration curve) and ~13.5 *μ*m depth. This layer was detached from the underlying substrate, after anodization, by applying a 120 mA/cm^2^ pulse (10 s) before being removed from the electrolyte; the free-standing membrane was then rinsed in methanol and air dried. Membranes were stored in ambient air for longer than 2 years which ensured complete native oxide growth prior to evaluation. To verify the sample morphology, the porosity of >50% and thickness of ~13 *μ*m of the *p*-Si membrane were estimated from the SEM images and optical characterization based on the transmittance *T*_0_ and reflectance *R*_0_ measurement and data analysis^23^. The samples used in this study do not show detectable luminescence, as p-silicon substrates are generally better suited to this purpose^41^. Instead, we used p+ substrates which are a better choice to obtain relatively thick and optically uniform membranes^28^. The average diameters of the pores and silicon interwoven wires were about 40 and 20 nanometer, respectively, and they do not have a strong quantum confinement for free carriers. We also investigated in our previous work the optical constants of the membrane which revealed that the real and imaginary parts of the complex effective dielectric function to be weakly dispersive around the values of ~3 and ~0.005, respectively^23^. To avoid confusion, we note that the recently published work on the charge carrier dynamics in *p*-Si was carried out on the samples with much higher porosity of >70% and using rather different wavelength in the 3.5–5 *μ*m range^24^. Hence, the results of that work should be compared with a perspective care as it is very likely that the probe wavelength and porosity affects the observed times of the carriers recombinations.
